# Genetic diversity and resistance profiles of MRSA in community and healthcare settings

**DOI:** 10.1186/s12866-026-05741-x

**Published:** 2026-11-12

**Authors:** May A. El-Antrawy, Mohamed Shohayeb, Lamis E. Elsawy, Heba A. Ramadan

**Affiliations:** Microbiology and Immunology Department, Faculty of Pharmacy, Delta University for Science and Technology, Gamasa, 11152 Egypt

**Keywords:** Methicillin-resistant *Staphylococcus aureus* (MRSA), Healthcare-associated infections, Community-acquired MRSA, Antibiotic resistance, ERIC fingerprinting

## Abstract

This study aimed to characterize and compare methicillin-resistant *Staphylococcus aureus* (MRSA) strains isolated from community members and healthcare workers (HCWs) in Egypt using antibiotic susceptibility testing, *mecA* gene detection, *SCCmec* typing, and ERIC-PCR fingerprinting. A cross-sectional study was conducted between September 2024 and January 2025, during which 450 specimens were collected from Mansoura University Hospitals and the local community, where 71 specimens (30.8%) were confirmed as MRSA. MRSA prevalence was significantly higher among HCWs, 62.5% (50/80), than among community members, 14% (21/150). In general, antibiotic resistance rates were higher in HCW strains. All isolates were resistant to cefoxitin and sensitive to vancomycin.

The methicillin-resistant phenotype of the 71 MRSA isolates was confirmed by positive *mecA* gene tests, with *SCCmec* Type III prevalent among healthcare workers and *SCCmec* Type IV predominating in the community. ERIC-PCR fingerprinting demonstrated the isolates’ genetic diversity. The dendrogram based on ERIC patterns showed distinct clusters between community and hospital strains, though with some overlap, suggesting possible transmission between the two populations.

In conclusion, MRSA strains from HCWs displayed broader antibiotic resistance and greater clonal similarity, while community strains were more diverse and less resistant. The presence of intermixed clusters between community-acquired (CA-MRSA) and (HCW-MRSA) suggested a possible bidirectional transmission pathway, underscoring the blurring of epidemiological boundaries.

These results underline the necessity of ongoing molecular surveillance and integrated infection control strategies to track the spread of MRSA in community and clinical settings.

**Supplementary Information:**

The online version contains supplementary material available at https://doi.org/10.1186/s12866-026-05741-x.

## Key points

• MRSA was significantly more prevalent among healthcare workers (62.5%) than among community individuals (14%), highlighting occupational risk.

• HCW MRSA strains exhibited markedly higher resistance rates to multiple antibiotics compared to community strains.

• Universal *mecA* gene detection, where all MRSA isolates carried the *mecA* gene.

• Dendrogram analysis revealed distinct clustering between community and healthcare MRSA strains, with some overlap suggesting possible cross-transmission.

**Supplementary Information:**

The online version contains supplementary material available at https://doi.org/10.1186/s12866-026-05741-x.

## Introduction

*Staphylococcus aureus* is a Gram-positive bacterium and a major cause of bacterial infections, with a strong potential to cause food poisoning and a variety of illnesses in people, such as abscesses, pneumonia, meningitis, endocarditis, and septicemia [1]. By expressing several virulence factors associated with its pathogenesis, this bacterium can adhere to surfaces and tissues, evade the immune system, and have detrimental toxic effects on the host [2]. These virulence factors are classified into two categories: cell surface-associated factors and secreted factors [2]. It is associated with high morbidity and mortality rates [3, 4].

The capacity of *S. aureus* to acquire antibiotic resistance is very well-known [5]. Based on antibiotic sensitivity, it is classified into methicillin-sensitive *Staphylococcus aureus* (MSSA) and methicillin-resistant *Staphylococcus aureus* (MRSA) [6]. In contrast to β-lactamase, the gene responsible for methicillin resistance (*mecA* gene) is situated on the chromosome and associated with “foreign DNA” that is present in resistant strains but not in susceptible ones [7]. According to transposon mutagenesis studies [8], the *mecA* gene, which *Song et al.* later sequenced, encodes PBP2a, a protein that confers methicillin resistance.

The *mecA* gene, carried on the Staphylococcal Cassette Chromosome *mec (SCCmec)*, encodes an alternative transpeptidase known as penicillin-binding protein 2a (PBP2a) [9]. Resistance is achieved through the significantly reduced affinity of PBP2a for β-lactam antibiotics, characterized by a lower rate of enzyme acylation and a prolonged acyl-enzyme half-life compared to native PBPs. These unique properties allow PBP2a to maintain essential cell wall cross-linking even in the presence of high antibiotic concentrations, ensuring bacterial survival [10, 11]. The emergence and spread of Methicillin-Resistant Staphylococcus aureus (MRSA), whose epidemiology differs greatly among individuals and geographic regions, pose a major concern to public health [12].

MRSA first emerged in the 1960s following its isolation from clinical specimens of hospitalized patients. MRSA has rapidly spread throughout communities all over the world since the early 1990s. However, the prevalence of MRSA infections has been reported to be reducing in the UK and several other developed countries [13].

Because of bacterial evolution and antibiotic abuse, MRSA is a multidrug-resistant “super bacterium” that is resistant to almost every class of antibiotics, including penicillins, cephalosporins, chloramphenicol, lincomycin, aminoglycosides, tetracyclines, macrolides, quinophthalones, sulfonamides, and rifampicin, due to bacterial evolution and the misuse of antibiotics. As a result, treating MRSA infections is extremely challenging and significantly increases morbidity and death. This emphasizes the urgent need for effective drugs to combat multidrug-resistant bacterial infections [14].

MRSA is now a major nosocomial and community-acquired infection despite its historical association with hospitals and healthcare environments [15]. Therefore, there is a pressing need for rapid and accurate diagnostic methods to identify MRSA strains. Such methods would provide crucial information on sensitivity to antimicrobial drugs, facilitating prompt and efficient treatment while halting its spread [12].

Epidemiologically, MRSA strains fall into two main categories: community-associated MRSA (CA-MRSA) and healthcare-associated MRSA (HA-MRSA). CA-MRSA infections typically occur in healthy individuals with no recent healthcare exposure, while HA-MRSA infections are frequently linked to hospitalization, invasive operations, or contact with healthcare surroundings [16]. These two groups differ not only in their resistance profiles but also in the types of *SCCmec* they harbor and their virulence determinants [17]. However, many reports have highlighted an increasing overlap between the two reservoirs, raising concerns about the spread of virulent and multidrug-resistant clones across community and healthcare boundaries.

Molecular typing methods like *SCCmec* typing and *mecA* gene detection are frequently used to comprehend the genetic diversity and spreading patterns of MRSA. The *mecA* gene is a key marker for methicillin resistance, while *SCCmec* typing provides insights into the evolutionary and epidemiological origin of MRSA strains [7]. Additionally, a quick and affordable genotyping technique that shows the genetic relatedness of MRSA isolates and supports outbreak investigation and source tracking is Enterobacterial Repetitive Intergenic Consensus-PCR (ERIC-PCR) fingerprinting [18].

Despite the global shift in MRSA epidemiology and the success of infection control programs in several developed nations, the molecular landscape of MRSA in developing regions, particularly within the Dakahlia Governorate of Egypt, remains insufficiently characterized. Current literature lacks comprehensive, updated comparative analyses between healthcare-associated and community-acquired MRSA lineages using both phenotypic resistance profiles and genotypic markers like the mecA gene. This data deficit hinders the development of evidence-based local antibiotic stewardship and targeted infection control policies, as the specific reservoirs and transmission dynamics between hospital staff and the public remain unclear.

In this study, we aimed to characterize MRSA isolates recovered from community individuals (CA-MRSA) and healthcare workers (HCW-MRSA) in Dakahlia Governorate, Egypt, by determining their antibiotic susceptibility profiles, detecting the presence of the *mecA* gene, performing SCC*mec* typing, and assessing their genetic relatedness using ERIC-PCR fingerprinting. The resulting dendrogram provides valuable information about the diversity and potential transmission routes of MRSA strains between the two populations, contributing to a better understanding of MRSA epidemiology in Egypt.

## Materials and methods

### Collection of specimens

Between September 2024 and January 2025, a total of 450 commensal MRSA isolates were collected from several sources at Mansoura University Hospitals. These included samples obtained from healthcare workers (doctors, nurses, laboratory technicians, and housekeeping staff) and community members (carriage samples). The specimens were collected using a consecutive sampling method over a five-month period. This sample size was determined by the inclusion of all eligible participants and clinical isolates available at the study sites during the specified timeframe, providing a comprehensive snapshot of MRSA prevalence in the Dakahlia Governorate. Specimens were collected using sterile transport swabs pre-moistened with sterile saline from the nostrils and the oropharynx. For the nasal samples, a single swab was rotated five times in each nostril. For throat samples, standardized swabbing of the tonsillar area was performed. For purification and identification, all specimens were transported right away to the Microbiology and Immunology Laboratory at Delta University.

### Isolation and identification of clinical isolates

All samples were streaked on mannitol salt agar (Oxoid, Hampshire, UK) and incubated at 37 °C for 24–48 h. According to [19], the characteristic yellow colonies were selected and further identified through Gram staining and biochemical tests [19]. The confirmed *S. aureus* isolates were kept on agar slants at 4 °C. Isolates were stored in tryptone soya broth (Oxoid, Hampshire, UK) with 20% glycerol at -20°C [20, 21] for long-term storage.

### Antimicrobial susceptibility testing

In accordance with the criteria of Clinical and Laboratory Standards Institute guidelines (CLSI, 2025) [20], antimicrobial susceptibility testing was performed using the standard disk diffusion method. 30 µg cefoxitin discs were used to screen *S. aureus* isolates to primarily detect MRSA. Antimicrobial disks were obtained from Oxoid (Hampshire, UK) and belonged to various classes, as shown in Table 1. Test tubes containing bacterial cultures were incubated at 37 °C overnight and adjusted to a turbidity of 0.5 McFarland standards. The surfaces of the dried Mueller‒Hinton agar plates (Oxoid, Hampshire, UK) were inoculated in three directions via a cotton swab, and the antibiotic disks were placed with alcohol-filled fine-pointed forceps. The plates were incubated in an inverted position at 37 °C for 16–18 h. Inhibition zones were measured and categorized as resistant (R), intermediate (I), or sensitive (S) on the basis of CLSI guidelines.

**Table 1:** Panel of antimicrobial agents, disc concentrations, and abbreviations used for susceptibility testing of MRSA isolates

Antibiotic	Abbreviation	Antimicrobial Class	Disc Content (µg)
Cefoxitin,	FOX	β-lactam drug	30
Vancomycin	VA	Glycopeptides	30
Levofloxacin	LEV	Fluoroquinolones	5
Lomifloxacin	LOM		10
Gentamicin	GM	Aminoglycoside	10
Tetracycline	TE	Tetracyclines	30
Imipenem	IPM	Carbapenems	10
Erythromycin	E	Macrolides	15
Clarithromycin	CLR		15
Co-trimoxazole	SXT	Folic acid inhibitors	1.25/23.752

### Preparation of DNA by the rapid DNA extraction (Boiling) method^22^

A 0.2 ml sterile PCR tube was filled with 100 µL of sterile nuclease-free water, and one or two fresh, pure S. aureus colonies were suspended in it. After heating the suspension for ten minutes at 95 °C in a thermocycler (FPROGO2D, Techne LTD, Oxford Cambridge, U.K.), it was centrifuged for five minutes at 5000 rpm. The resulting supernatant, containing the extracted DNA, was stored at -20 °C for further use.

### Gene amplification and visualization^23^

Using oligonucleotide primers (QIAGEN, USA) specified in Table 2, PCR was used to amplify particular *S. aureus* genes, such as *mecA* and *SCCmecA*. The following reaction mixture was used to perform the amplification: A total of 25 µL per reaction is required, which includes 12.5 µL of DreamTaq Green PCR Master Mix (2X) (ThermoScientific, UK), 3 µL of bacterial DNA, 1 µL of each primer (10 µM), and 7.5 µL of nuclease-free water. A denaturation stage at 94 °C for 4 min kicked off the amplification procedure for all genes. This was followed by 30 amplification cycles and a final extension at 72 °C for 2 min.

Each cycle for the *mecA* gene comprised 30 s of denaturation at 94 °C, 30 s of annealing at 58 °C, and 1 min of extension at 72 °C. The cycles for the *SCCmecA* gene included 30 s of denaturation at 94 °C, 60 s of annealing at 55 °C, and 40 s of extension at 72 °C.

The PCR products were separated through the horizontal gel electrophoresis technique using a 1.5% agarose gel, at 120 V for 45 min, stained with ethidium bromide, and visualized by a UV transilluminator (Model UV-TM-25–230 V, Hoefer, U.S.A.) at 312 nm.

**Table 2:** List of oligonucleotide primers used for detection of *mecA* and SCC*mec*

Gene	Type*	Nucleotide Sequence (5’ to 3’)	Product Size (Bp)	SCC*mecA* Types					References
				I	II	III	IV	V	
*mecA*	Fw	(AAA ATC GAT GGT AAA GGT TGG C)	532						[24]
	Rv	(AGT TCT GCA GTA CCG GAT TTG C )							
Scc*mec*	β	(ATTGCCTTGATAATAGCCYTCT)	937		X		X		[25]
	α3	(TAAAGGCATCAATGCACAAACACT)							
	ccrCF	(CGTCTATTACAAGATGTTAAGGATAAT*)*	518			X		X	
	ccrCR	(CCTTTATAGACTGGATTATTCAAAATAT)							
	1272F1	(GCCACTCATAACATATGGAA)	415	X			X		
	1272R1	(CATCCGAGTGAAACCCAAA)							
	5RmecA	(TATACCAAACCCGACAACTAC)	359					X	
	5R431	(CGGCTACAGTGATAACATCC*)*							

* Fw, forward primer; Rv, reverse primer; Bp, Base pair

### Molecular typing of MRSA isolates by enterobacterial repetitive intergenic consensus-PCR (ERIC-PCR)

MRSA isolates were fingerprinted by ERIC-PCR using the following primer: ERIC 1 (5′-ATGTAAGCTCCTGGGGATTCAC-3′). The DNA amplification process was performed in a volume of 50 µL, including 2 U Go Taq DNA polymerase (Fermatas), 2.5 mM MgCl₂ (Invitrogen), 0.25 mM each deoxynucleotide triphosphate, 100 ng of DNA template, and 0.5 µM primers, while the remaining volume was filled up with PCR-grade water. The PCR program used was as follows: denaturation at 95 °C for 5 min., followed by 35 cycles of denaturation at 94 °C for 30 s, annealing at 42 °C for 40 s, and extension at 72 °C for 5 min., and then a single final stage at 72 °C for 10 min [23].

### Dendrogram and phylogenetic relationships

The banding patterns of the PCR products were resolved on a 1.5% (w/v) agarose gel at 80 V for 90 min. A negative control (DNase-free water without DNA template) was included in every PCR run to ensure the absence of contamination. The molecular weight of the amplified fragments was determined by comparison with a 100 bp DNA ladder (GeneRuler, Thermo Scientific), and amplicons were identified based on the expected product sizes as described in Table 2. The dendrogram was created by the unweighted pair group method with arithmetic mean (UPGMA) using the Excel Dendrogram Generator (https://doi.org/10.5281/zenodo.15769051). It was constructed using the Dice coefficient with a 1% tolerance limit and 1% optimization. Cluster relatedness of collected isolates with ≥ 70% similarity was considered to indicate an identical pattern type [26].

### Statistical analysis

Data analysis was conducted using GraphPad Prism software. Chi-square test was applied to compare the resistance rates of MRSA isolates from community and healthcare workers. Significance was set to * *p* < 0.05, ** *p* < 0.01, and *** *p* < 0.001.

## RESULTS

### Distribution of MRSA

Out of the 450 collected specimens, 230 (51.1%) isolates were identified as *S. aureus*. Out of these 230 isolates of *S. aureus*, 71 specimens were identified as MRSA (30.8%). The distribution of MRSA among HCWs and community members was 62.5% (50/80) and 14% (21/150), respectively.

### Antimicrobial susceptibility testing

The antimicrobial resistance rates of MRSA isolates were identified according to the Clinical and Laboratory Standards Institute (CLSI), which provided standardized guidelines for interpreting disk diffusion susceptibility through the diameter of inhibition zones (measured in millimeters) that categorized bacterial isolates as susceptible (S), intermediate (I), or resistant (R) to the tested antibiotics.

**Fig. 1:**
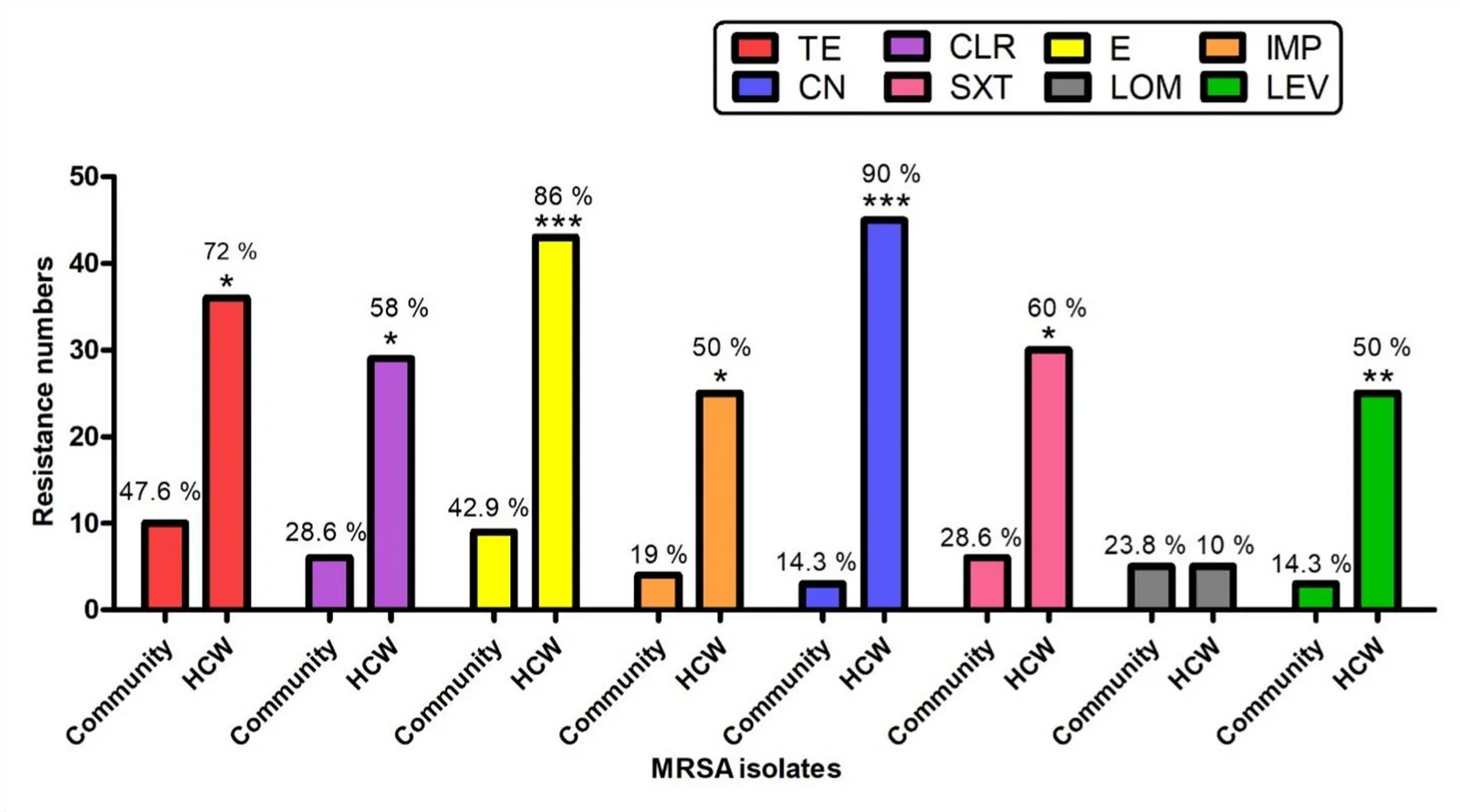
Comparative rate of resistance between the MRSA isolated from community and healthcare workers exposed to different classes of antibiotics

The data represented in Table 3; Fig. 1 compared the antibiotic resistance rates of Methicillin-Resistant *Staphylococcus aureus* (MRSA) strains obtained from community individuals versus those from healthcare workers. One noteworthy finding is that MRSA strains isolated from HCWs generally exhibit noticeably greater rates of resistance to almost all tested antibiotics compared to community-acquired isolates. Two notable exceptions are cefoxitin, to which both groups exhibit 100% resistance (as expected for MRSA), and vancomycin, to which both groups show 0% resistance, suggesting that those strains are vancomycin sensitive.

Antimicrobial resistance profiles differed significantly between the two study groups, with HCW isolates exhibiting markedly higher resistance across nearly all tested agents (Table 3). The most pronounced disparities were observed for gentamicin and erythromycin (*p* < 0.001), where resistance in HCW strains reached 90% and 86%, respectively, compared to much lower rates in community isolates (14.3% and 42.9%). Significant differences were also noted for levofloxacin (*p* < 0.01), as well as tetracycline, trimethoprim/sulfamethoxazole, clarithromycin, imipenem, and ciprofloxacin (*p* < 0.05). Lomefloxacin was the only antibiotic that did not show a statistically significant difference between the two groups. Notably, while community strains were generally more susceptible, they maintained considerable resistance levels to tetracycline (47.6%) and erythromycin (42.9%).

**Table 3:** Comparison of antimicrobial resistance profiles between community-acquired (*n* = 21) and healthcare-associated (*n* = 50) MRSA isolates

Antibiotics	Resistant		Intermediate		Sensitive	
	Community Isolates No. (%)	HCWs Isolates No. (%)	Community Isolates No. (%)	HCWs Isolates No. (%)	Community Isolates No. (%)	HCWs Isolates No. (%)
**Cefoxitin (FOX)**	21 (100%)	50(100%)	-	-	-	-
**Vancomycin (VA)**	-	-	-	-	21(100%)	50 (100%)
**Tetracycline (TE)**	10 (47.6%)	36 (72%)	-	2 (4%)	11(52.4%)	12 (24%)
**Clarithromycin (CLR)**	6 (28.6%)	29 (58%)	-	1 (2%)	15 (71.4%)	20 (60%)
**Erythromycin (E)**	9 (42.9%)	43 (86%)	5 (23.8%)	1 (2%)	7 (33.3%)	6 (12%)
**Imipenem (IMP)**	4 (19%)	25 (50%)	-	-	17 (81%)	25 (50%)
**Gentamicin (CN)**	3 (14.3%)	45 (90%)	-	-	18 (85.7%)	5 (10%)
**Trimethoprim-Sulfamethoxazole (SXT)**	6 (28.6%)	30 (60%)	3 (14.3%)	-	12 (57.1%)	20 (40%)
**Lomefloxacin (LOM)**	5 (23.8%)	5 (10%)	-	-	16 (76.2%)	45 (90%)
**Levofloxacin (LEV)**	3 (14.3%)	25 (50%)	2 (9.5%)	2 (4%)	16 (76.2%)	23 (46%)

Chi-square test was used to compare the MRSA isolates from the community and HCW. *, **, *** significant from community group at *p* < 0.05, *p* < 0.01, and *p* < 0.001 respectively. TE, HCW, Healthcare workers; Tetracycline; CLR: Clarithromycin; E, Erythromycin; IMP, Imipenem; CN, Gentamicin; SXT, Trimethoprim-Sulfamethoxazole; LOM, Lomefloxacin; LEV: Levofloxacin.

### Detection of the *mecA* gene

The molecular detection of the *mecA* gene is evidenced by a characteristic 532 bp amplicon. The *mecA* gene was successfully identified in all 21 MRSA isolates collected from community individuals and in all 50 MRSA isolates collected from healthcare workers. The prevalence of the *mecA* gene, genetically, supported the methicillin resistance phenotype observed in these *S. aureus* isolates, classifying them as MRSA and underpinning their resistance to the beta-lactam antibiotic cefoxitin, to which both MRSA groups demonstrated 100% resistance, as mentioned before.

### Detection of SCC*mec* genes

A total of 71 MRSA isolates were successfully typed for their staphylococcal cassette chromosome *mec (SCCmec)* element. The distribution of *SCCmec* types varied notably between isolates from community-acquired infections and those from healthcare worker carriage. Among the twenty-one community-acquired isolates, *SCCmec* Type IV was the most predominant, identified in 66.6% (14/21) of strains. *SCCmec* Type V was the second most common, accounting for 19% (4/21) of isolates. In contrast, among the 50 isolates from healthcare workers, *SCCmec* Type III was the most frequently detected type at 50% (25/50), followed by *SCCmec* Type IV at 42% (21/50). The remaining isolates were distributed among *SCCmec* Type II at 20% (10/50) and *SCCmec* Type V at 14% (7/50). These findings illustrated a clear epidemiological divergence, with community strains being dominated by Type IV while healthcare workers harbor a diverse mix of genotypes.

### DNA fingerprinting analysis by ERIC-PCR

The fingerprints obtained from a PCR assay with ERIC primer of MRSA isolates showed a DNA banding profile consisting of amplified bands from 75 to 5000 bp in size. Based on the binary data of these isolates, a dendrogram was constructed. Isolates were considered to have the same pattern upon a similarity level of ≥ 70%. The dendrogram presented illustrates the hierarchical clustering of 71 MRSA isolates. The dendrogram showed a degree of separation between community-associated MRSA (CA-MRSA) and healthcare-associated MRSA (HA-MRSA), although some overlap exists, suggesting potential genetic relatedness and transmission across the two environments.

Specifically, two major clusters can be observed: one predominantly composed of hospital isolates (highlighted in orange) and another cluster enriched with community isolates (highlighted in blue). The presence of intermixed clusters—where some community isolates cluster more closely with hospital strains and vice versa—suggested a possible bidirectional transmission pathway.

Of particular note is the clustering of community isolates, such as C6 and C14, nearer to hospital strains. This may reflect either the adaptation of CA-MRSA strains within healthcare settings or the acquisition of HA-MRSA strains by community members, possibly through healthcare exposure or indirect contact with colonized personnel. Notably, isolate C14 represents an ancestral lineage to this cluster, suggesting that this successful hospital-circulating clone likely originated from a strain endemic in the community. More critically, some community isolates were found to be clonally related to HCW isolates like (C16-H5) and (C18-H29), providing strong evidence of a recent and active transmission event bridging the two environments. This finding demonstrates that the boundaries between community and hospital MRSA are highly porous and that successful community-associated lineages can be introduced into healthcare settings, where they can establish themselves and cause nosocomial outbreaks.

The average linkage distances also provide insight into the genetic diversity among isolates. The relatively tight clustering of some subgroups (e.g., C3-C5 and C2–C4) indicates a high degree of similarity.

Overall, the clustering results support the hypothesis that MRSA transmission in Egypt is not strictly compartmentalized. The overlapping patterns highlight the need for integrated infection control measures that encompass both community and healthcare settings. These findings advocate for enhanced surveillance, particularly of healthcare workers, who may serve as potential reservoirs and vectors of transmission between the two environments.

## Discussion

Our study provided a detailed molecular analysis of the MRSA strains circulating among community members and healthcare workers (HCWs) in the Dakahlya Governorate, Egypt. It also revealed a complex interaction between community- and hospital-associated lineages and provided strong molecular evidence for the transmission of MRSA. Previous studies suggested an increase in MRSA infections in developing countries [27]. In comparison to the USA and Europe, the total prevalence of MRSA in this study was 30.8.% (71/230), which is high [28–30]. In addition, its incidence was relatively higher among HCWs (62.5%) compared to CA-MRSA (14%). However, Egypt lacks a nationwide surveillance infrastructure that would allow data collected in various cities to be extrapolated to the entire country. According to reports, hospitalized patients in Cairo and Alexandria, two major Egyptian cities, had a general MRSA prevalence rate of 50–82%, while the less populous city of Minia in southern Egypt had a lower rate of 24% [31]. While the prevalence of community-acquired-MRSA varies from 19 to 47% [32], which came in agreement with [23].

The study’s remarkably elevated MRSA incidence could be explained by a number of factors, including the overuse of antibiotics due to their wide availability, the prescription of antibiotics for viral infections, and the lack of quick and precise techniques for identifying carriers. On the other hand, isolation of colonized or infected patients and the execution of entrance screening cultures for MRSA are two of the primary infection control strategies for preventing the spread of nosocomial MRSA infection [33]. It appears that Mansoura hospitals do not strictly follow this approach. Additionally, there is a patient escort system in Egypt’s tertiary hospitals. In this instance, the patient’s room is occupied by close relatives. In order to prevent CA-MRSA from spreading throughout hospitals, patient companions should also be checked for MRSA. Thus, it is not unexpected that 62.5% of MRSA isolates were found in healthcare workers. According to the European Centre for Disease Prevention and Control (ECDC) and the Centers for Disease Control and Prevention (CDC) [34], the seventy-one recovered MRSA isolates were multidrug resistant in terms of antibiotic susceptibility testing, and they were all cefoxitin-resistant, which was consistent with [23]. The HCW-MRSA revealed significant levels of gentamicin, erythromycin, and tetracycline resistance rates of 90%, 86%, and 72%, respectively. This may be attributed to class I integrons, which have been associated with the resistance of staphylococci to several antibiotics and are common in MRSA isolates from Egypt [35].

On the other hand, CA-MRSA were generally less resistant to the various antibiotic classes that have been studied; they still showed significant resistance to some drugs, such as erythromycin and tetracycline, which had the highest resistance rates (47.6% and 42.9%, respectively). This confirms a previous report of a low resistance rate for CA-MRSA in Mansoura [35].

However, all MRSA isolates were sensitive to vancomycin. Consequently, culture sensitivity tests should be utilized to guide the use of vancomycin in treating MRSA isolates. According to previous research done in Egypt, the prevalence of VRSA was 23.45% (Lamis) and 21.7% [36]. The rising misuse of vancomycin to treat MRSA infections would be the cause of the former observations, which show a concerning rise in vancomycin resistance in several parts of Egypt, limiting this treatment option.

Our study indicated that HCW-associated MRSA strains possess a more formidable resistance profile, likely reflecting the higher antibiotic exposure and selection pressure within healthcare environments. This highlights the greater therapeutic challenge posed by MRSA infections acquired in healthcare settings and underscores the importance of targeted antibiotic stewardship and infection control measures.

Isolates from HCWs were dominated by *SCCmec* Type III. As expected, this genotype and MDR phenotype showed a high correlation [37, 38]. Most of these isolates showed resistance to several kinds of antibiotics, such as macrolides (erythromycin and clarithromycin), fluoroquinolones (levofloxacin and lomifloxacin), and aminoglycosides (gentamicin). This standard HA-MRSA profile highlights the occupational risk encountered by healthcare workers [39]. The frequency of *SCCmec* types found in our community isolates is consistent with Egypt’s and the Middle East’s known epidemiological landscape. *SCCmec* Type IV’s pronounced dominance is in line with earlier Egyptian research that has consistently shown it to be the main cause of Community-Acquired (CA-MRSA) infection [40]. The significant prevalence of *SCCmec* Type V as the second most common genotype mirrors regional MRSA trends in Egypt, where meta-analyses and hospital surveillance studies have reported Type V rates ranging from 60 to 75%, and recent data show it to be the predominant *SCCmec* type (~ 34%) in tertiary-care settings [41, 42]. Moreover, among outpatient skin and soft-tissue infections, 50% of CA-MRSA isolates carried *SCCmec* V, highlighting its prominent role in community-associated disease clusters [43]. This pattern likely reflects the successful establishment and dispersion of these small, highly mobile *SCCmec* cassettes, which facilitate efficient horizontal transfer and colonization—thereby increasing SSTI frequency in outpatient settings across the Egyptian population.

Recent regional and international studies further support the epidemiological patterns observed in our findings. For example, Sedaghat et al. (2018) reported the emergence of MRSA clones ST639-*SCCmec* III and ST343-*SCCmec* III in a teaching hospital in Iran, highlighting the predominance of *SCCmec* type III in healthcare-associated settings and its association with multidrug resistance. These findings are consistent with our observation that *SCCmec* type III was dominant among healthcare worker isolates, reinforcing the established link between type III and hospital-adapted MRSA lineages [44].

On a global scale, our findings were in agreement with the well-documented pandemic of CA-MRSA, which is largely driven by strains carrying *SCCmec* IV and V [45]. These strains are often characterized by enhanced virulence, such as the presence of Panton-Valentine Leukocidin (*PVL*), and susceptibility to non-β-lactam antibiotics. On the other hand, our HCW isolates’ high frequency of *SCCmec* Type III matches the traditional Hospital-Acquired (HA-MRSA) profile. Worldwide, endemic hospital clones are characterized by their sizable, multidrug-resistant cassette, which conforms to their ongoing exposure to an environment high in antibiotics [46].

Moreover, clusters of MRSA isolates with ≥ 90% similarity were found in the ERIC-PCR dendrogram, suggesting clonal linkages among strains of healthcare workers. Community isolates, on the other hand, displayed lower inter-isolate similarity and higher genetic diversity, indicating multiple and independent origins of acquisition. Possible transmission across healthcare and community settings is seen in the existence of mixed clusters.

Our dendrogram analysis showed phylogenetic evidence of transmission between community and hospital environments, which may be the study’s most important discovery. A “smoking gun” for an active transmission event is the clonal link between community and HCW isolates (C16-H5) and (C18-H29), which were genetically nearly similar (Jaccard distance 0.15). This discovery calls into question the strict distinction between CA- and HA-MRSA. It is highly likely that community clones are not only being brought into the hospital but can also spread among the employees, thereby turning into nosocomial strains. Because of this “bridging” phenomenon, healthcare workers are in a crucial position to spread strains both from the hospital to the community and vice versa ([47]; Elsayed et al., 2023).

Additionally, recent epidemiological investigations have emphasized the ongoing genetic diversification of MRSA clones and the increasing overlap between community- and hospital-associated strains [48]. These studies underscore the dynamic evolution of *SCCmec* elements and support our observation of intermixed ERIC-PCR clusters, suggesting bidirectional transmission and the erosion of traditional epidemiological boundaries between CA-MRSA and HA-MRSA [39, 47, 49].

This finding aligns with previous epidemiological studies that report the infiltration of hospital-associated clones into the community and vice versa due to increased contact between populations, healthcare access, and antibiotic pressure [50, 51].

## Conclusion

The study’s findings support Egypt’s dual epidemiology of MRSA, with Type III prevalent among healthcare workers and SCCmec Type IV predominating in the community. More significantly, it provides clear molecular proof that the lines between these two reservoirs are fundamentally blurred. Future studies are warranted to better monitor these transmission dynamics with greater precision and inform public health strategies aimed at curbing the spread of MRSA in both the community and our hospitals. The presence of intermixed clusters—where some community isolates cluster more closely with hospital strains and vice versa—suggested a possible bidirectional transmission pathway, underscoring the blurring epidemiological boundaries between CA-MRSA and HCW-MRSA.

These results underline the necessity of ongoing molecular surveillance and integrated infection control strategies to track the spread of MRSA in community and clinical settings. We recommend the establishment of mandatory, periodic screening programs for HCWs to identify asymptomatic reservoirs of *mecA*-positive strains. For those identified as persistent carriers, targeted decolonization protocols—utilizing 2% mupirocin nasal ointment or chlorhexidine gluconate washes—should be enforced to break the cycle of transmission. Furthermore, these measures must be supported by rigorous hand hygiene audits and strict adherence to contact precautions, including the use of personal protective equipment (PPE) in high-prevalence units, to safeguard both the staff and vulnerable patient populations.

Beyond the hospital environment, our findings underscore the urgency of comprehensive antibiotic stewardship and public health initiatives to curb the rising resistance in the community. Promoting diagnostic precision through the early adoption of rapid molecular testing, such as *mecA* PCR, is essential for guiding appropriate empirical therapy and preventing the misuse of “last-resort” antibiotics. On a broader scale, local health authorities should implement formulary restrictions and launch public awareness campaigns to educate community members on the dangers of self-medication and over-the-counter antibiotic use. By bridging the gap between molecular surveillance and clinical practice, these dual strategies can effectively mitigate the spread of resistant staphylococcal lineages in both healthcare and community reservoirs.

Finally, while this study provides significant insights into local MRSA epidemiology, it is limited by the lack of a formal pre-study power analysis. However, the inclusion of 450 samples from diverse community and hospital sources provides a robust preliminary dataset for the region, serving as a foundation for future larger-scale longitudinal studies. We aim to address this in future investigations by expanding the scope and scale of sample collection and analysis.

## Supplementary information

Below is the link to the electronic supplementary material.

Supplementary Material 1

## Data Availability

All data generated or analyzed during this study are included in this published article [and its supplementary information files].
