# Supplementary material for: Genetic diversity and resistance profiles of MRSA in community and healthcare settings
Source: BMC Microbiol. 2026 Nov 12;26:1003. doi: 10.1186/s12866-026-05741-x (PMC13761016; doi:10.1186/s12866-026-05741-x)
Supplement: Supplementary file 1 [file 12866_2026_5741_MOESM1_ESM.docx]

**Genetic Diversity and Resistance Profiles of MRSA in Community and Healthcare Settings**

**May A. El-Antrawy*, Mohamed Shohayeb, Lamis E. Elsawy, Heba A. Ramadan**

Microbiology and Immunology Department, Faculty of Pharmacy, Delta University for Science and Technology, Gamasa 11152, Egypt.

**Table S1:** **Susceptibility Patterns of MRSA Isolates Collected from Community Individuals.**

| **Stain No.** | **FOX** | **VA** | **TE** | **CLR** | **E** | **IMP** | **CN** | **SXT** | **LOM** | **LEV** |
| --- | --- | --- | --- | --- | --- | --- | --- | --- | --- | --- |
| **C1** | R | S | R | R | R | S | S | S | S | S |
| **C2** | R | S | S | S | I | S | S | S | S | S |
| **C3** | R | S | S | S | S | S | S | S | S | S |
| **C4** | R | S | R | S | S | S | S | S | S | S |
| **C5** | R | S | R | R | R | R | S | R | S | S |
| **C6** | R | S | S | S | I | S | S | R | S | S |
| **C7** | R | S | S | S | S | S | S | S | S | S |
| **C8** | R | S | R | R | R | R | S | S | S | S |
| **C9** | R | S | S | S | I | S | S | R | S | S |
| **C10** | R | S | R | S | R | R | S | S | S | S |
| **C11** | R | S | S | S | R | S | S | I | R | I |
| **C12** | R | S | S | S | I | S | S | S | S | S |
| **C13** | R | S | S | S | I | S | R | S | S | S |
| **C14** | R | S | R | R | R | R | S | S | S | S |
| **C15** | R | S | S | S | S | S | S | R | R | I |
| **C16** | R | S | R | S | S | S | R | S | S | S |
| **C17** | R | S | S | S | S | S | S | R | R | R |
| **C18** | R | S | S | S | S | S | S | S | S | S |
| **C19** | R | S | S | S | S | S | S | I | S | S |
| **C20** | R | S | S | S | S | S | S | I | R | R |
| **C21** | R | S | R | S | S | S | R | S | S | S |

**Table S2:** **Susceptibility Patterns of MRSA Isolates Collected from Health Care Workers (HCWs).**

| **Stain No.** | **FOX** | **VA** | **TE** | **CLR** | **E** | **IMP** | **CN** | **SXT** | **LOM** | **LEV** |
| --- | --- | --- | --- | --- | --- | --- | --- | --- | --- | --- |
| **H1** | R | S | R | R | R | S | R | R | S | R |
| **H2** | R | S | R | R | R | S | R | R | S | R |
| **H3** | R | S | R | S | R | S | R | R | S | S |
| **H4** | R | S | R | R | R | S | R | R | S | R |
| **H5** | R | S | R | S | I | S | R | R | S | S |
| **H6** | R | S | R | S | R | R | R | S | S | S |
| **H7** | R | S | R | R | R | S | R | S | S | S |
| **H8** | R | S | I | S | R | S | R | S | S | S |
| **H9** | R | S | R | R | R | R | R | R | S | R |
| **H10** | R | S | R | R | R | R | R | R | S | R |
| **H11** | R | S | R | R | R | S | R | S | R | R |
| **H12** | R | S | S | S | R | R | R | R | S | I |
| **H13** | R | S | R | R | R | R | R | S | S | S |
| **H14** | R | S | S | R | R | S | R | S | S | R |
| **H15** | R | S | R | R | R | R | R | R | S | R |
| **H16** | R | S | R | R | R | R | R | S | S | R |
| **H17** | R | S | S | S | S | S | R | S | S | S |
| **H18** | R | S | S | S | R | R | R | R | R | R |
| **H19** | R | S | R | S | R | R | R | S | S | R |
| **H20** | R | S | R | S | R | S | S | S | S | S |
| **H21** | R | S | R | S | R | R | S | R | S | S |
| **H22** | R | S | S | R | S | R | R | R | S | R |
| **H23** | R | S | R | I | R | S | R | R | R | I |
| **H24** | R | S | R | S | R | R | R | R | S | S |
| **H25** | R | S | R | R | S | S | R | S | S | S |
| **H26** | R | S | R | R | R | R | S | R | S | R |
| **H27** | R | S | S | R | R | R | R | R | S | R |
| **H28** | R | S | I | R | R | S | R | R | S | S |
| **H29** | R | S | S | R | R | R | R | R | S | R |
| **H30** | R | S | R | S | R | R | R | R | S | R |
| **H31** | R | S | S | R | R | R | R | R | S | S |
| **H32** | R | S | R | R | R | S | R | R | S | R |
| **H33** | R | S | R | S | S | R | R | S | S | R |
| **H34** | R | S | R | S | R | R | R | R | S | S |
| **H35** | R | S | S | R | R | S | R | S | S | R |
| **H36** | R | S | R | R | R | S | R | R | S | S |
| **H37** | R | S | R | S | S | R | R | S | S | S |
| **H38** | R | S | R | R | R | R | R | R | R | R |
| **H39** | R | S | S | S | R | R | R | R | S | S |
| **H40** | R | S | S | R | R | S | R | S | S | R |
| **H41** | R | S | R | S | S | S | S | S | S | S |
| **H42** | R | S | R | R | R | R | R | S | S | R |
| **H43** | R | S | R | R | R | S | R | S | S | S |
| **H44** | R | S | S | S | R | R | S | R | S | S |
| **H45** | R | S | R | R | R | S | R | S | S | S |
| **H46** | R | S | R | R | R | R | R | S | S | S |
| **H47** | R | S | R | S | R | S | R | R | S | R |
| **H48** | R | S | R | R | R | S | R | R | S | R |
| **H49** | R | S | R | R | R | S | R | R | R | S |
| **H50** | R | S | R | S | R | S | R | R | S | R |

|  | Community | HCWs |
| --- | --- | --- |
| FOX | 100% | 100% |
| VA | 0% | 0% |
| TE | 47.6% | 72% |
| CLR | 28.6% | 58% |
| E | 42.9% | 86% |
| IMP | 19% | 50% |
| CN | 14.3% | 90% |
| SXT | 28.6% | 60% |
| LOM | 23.8% | 10% |
| LEV | 14.3% | 50% |

**Figure S1: Comparison between** **rate of resistance of the MRSA isolated from community and healthcare workers exposed to different classes of antibiotics**

**Figure S2: Molecular typing of 71 MRSA isolates using ERIC-PCR.**

a) Dendrogram analysis: Genetic relatedness of the isolates based on UPGMA clustering using the Dice coefficient. The vertical red line indicates the 70% similarity threshold used to define major clusters. Strains with $\ge$ 70% similarity were considered genetically related.

(b) Representative Agarose Gel Electrophoresis: DNA fingerprinting profiles generated using the ERIC 1 primer.

Lane M: 100 bp DNA ladder (molecular weight marker).

Lane (-ve): Negative control (PCR mixture without DNA template).

Electrophoresis was performed on a 1.5% (w/v) agarose gel at 80V for 90 minutes and visualized under UV light after ethidium bromide staining.

# **Figure S3: Gel electrophoresis profile of the *mecA* gene for some representative CA-MRSA and HCW-MRSA.**

# **M: molecular size marker (100 bp DNA ladder)**

**Figure S4: Gel electrophoresis profile of the SCC*mec* genes for some representative CA-MRSA isolates**

M: molecular size marker (100 bp DNA ladder)

**Figure S5: DNA fingerprinting by ERIC-PCR.**

**A) for some representative community isolates**

**Figure S6: DNA fingerprinting by ERIC-PCR.**

**B) for some representative community isolates**
